# Detection of soluble EpCAM (sEpCAM) in malignant ascites predicts poor overall survival in patients treated with catumaxomab

**DOI:** 10.18632/oncotarget.4496

**Published:** 2015-07-10

**Authors:** Andreas Seeber, Ioana Braicu, Gerold Untergasser, Mani Nassir, Dominic Fong, Laura Botta, Guenther Gastl, Heidi Fiegl, Alain Zeimet, Jalid Sehouli, Gilbert Spizzo

**Affiliations:** ^1^ Department of Haematology and Oncology, Innsbruck Medical University, Innsbruck, Austria; ^2^ Tyrolean Cancer Research Institute, Innsbruck, Austria; ^3^ Oncotyrol – Center for Personalized Cancer Medicine, Innsbruck, Austria; ^4^ European Competence Center for Ovarian Cancer, Charité Berlin, Berlin, Germany; ^5^ Haemato-Oncological Day Hospital, Hospital of Merano, Merano, Italy; ^6^ Evaluative Epidemiology Unit, Fondazione IRCSS “Istituto Nazionale dei Tumori”, Milan, Italy; ^7^ Department of Gynaecology and Obstetrics, Innsbruck Medical University, Innsbruck, Austria

**Keywords:** EpCAM, soluble EpCAM, catumaxomab, ascites, ovarian cancer

## Abstract

EpCAM is an attractive target for cancer therapy and the EpCAM-specific antibody catumaxomab has been used for intraperitoneal treatment of EpCAM-positive cancer patients with malignant ascites. New prognostic markers are necessary to select patients that mostly benefit from catumaxomab. Recent data showed that soluble EpCAM (sEpCAM) is capable to block the effect of catumaxomab *in vitro*. This exploratory retrospective analysis was performed on archived ascites samples to evaluate the predictive role of sEpCAM in catumaxomab-treated patients. Sixty-six catumaxomab-treated patients with an available archived ascites sample were included in this study and tested for sEpCAM by sandwich ELISA. All probes were sampled before treatment start and all patients received at least one catumaxomab infusion. Overall survival, puncture-free survival and time to next puncture were compared between sEpCAM-positive and -negative patients. We detected sEpCAM in ascites samples of 9 patients (13.6%). These patients showed a significantly shorter overall survival. The prognostic significance of sEpCAM in ascites was particularly strong in patients with ovarian cancer. Puncture-free survival and time to next puncture were not significantly different between sEpCAM-positive and -negative patients. We propose sEpCAM in malignant ascites as a potential predictive marker in cancer patients treated with catumaxomab. Prospective studies with larger patients samples are urgently needed to confirm these findings and studies testing dose-intensified catumaxomab in patients with sEpCAM-positive ascites should be envisaged.

## INTRODUCTION

The Epithelial cell adhesion antigen EpCAM (gene name TACSTD1) has been identified as tumour associated antigen on carcinomas of various origins. It rapidly emerged as an attractive target for specific immunologic approaches and the first monoclonal antibody that was ever used in patients with gastrointestinal tumours three decades ago was the EpCAM-directed monoclonal antibody 17-1A [1]. In adult humans, EpCAM is frequently expressed in normal epithelia [2]. In patients with carcinomas, an overexpression of this antigen is frequently observed. In primary and metastatic breast cancer, for example, it has been shown that EpCAM gene expression is increased up to 1,000-fold [3]. We have recently summarized the EpCAM expression rates in most human tumour entities [4] and we have shown that the expression in metastases usually reflects that of the primary tumour tissue. Soft-tissue tumours and all haematological neoplasms are usually EpCAM negative.

Of note, EpCAM appears to display oncologic features in certain *in vitro* cell line models. Hence, overexpression of EpCAM was associated with enhanced transcription of the proto-oncogene c-myc and the cell cycle proteins cyclin A and E [5]. Moreover, the proteolytic cleavage of the EpCAM molecule was shown to confer a mitogenic signal to the cell nucleus by shuttling the intracellular domain called EpICD to the nucleus and shedding the extracellular domain named EpEX to the extracellular space [6].

Paradoxically, despite the broad expression of EpCAM in different tumour tissues, most EpCAM-based targeting strategies has shown only limited efficacy [7]. In the last decades, monoclonal, bi-/tri-specific antibodies, vaccination strategies and toxin-conjugated antibodies have been used to target the EpCAM antigen. Currently, clinical trials are testing different immunotherapeutic approaches [8].

In 2009 the European Commission approved the first anti-EpCAM antibody named catumaxomab [9] for the treatment of malignant ascites in cancer patients with EpCAM-positive tumours. A phase II/III study [10] was conducted and data showed a significant clinical benefit in catumaxomab-treated patients. Catumaxomab is administered as an intraperitoneal infusion on days 0, 3, 7 and 10 at increasing doses of 10, 20, 50 and 150 μg, respectively. As the antibody is diluted in remnant ascites and concomitantly administered saline infusions with an expected total volume of ∼1, 000 mL, final concentration of the antibody in the peritoneal cavity probably ranges from ∼10 to 150 ng/mL. Importantly, quality of life was improved as puncture-free survival, time to next puncture and symptoms of ascites were significantly better in the treated than in the control cohort. Moreover, in gastric cancer patients, overall survival was significantly prolonged. However, the selection of patients that are candidates for catumaxomab treatment in daily clinical practice is difficult. Malignant ascites is often an end-stage situation and survival of these patients is usually very short [11]. In Italy for example, the regulatory agency for medical drugs (AIFA) reimburses treatment for patients with a life expectancy of more than 3 months. For this reason, it is mandatory to search for prognostic and predictive markers to better select patients that mostly benefit from this treatment.

Recently, we developed an EpCAM-specific ELISA system to detect soluble EpCAM (sEpCAM) in ascites [12]. We could show that detection of sEpCAM correlates with peritoneal carcinomatosis. Moreover, within a catumaxomab (antibody)-dependent cellular cytotoxicity (ADCC) assay we could show that sEpCAM is neutralizing the effect of catumaxomab already in a concentration of 1 ng/mL [12]. In this study we investigated the effect of sEpCAM amount in malignant-ascites in patients treated we catumaxomab to corroborate our findings in an *in vivo* approach.

## RESULTS

### Patients` characteristics and levels of sEpCAM in ascites

At the time of last clinical follow-up (January 2014), 47 (71.2%) patients out of the total group had died. The median overall survival time for the entire cohort was 143 days (range, 8–1, 884). Most patients (*n* = 43) were diagnosed with epithelial ovarian cancer (65.2%). All other patients had non-ovarian cancer, which included gastric cancer (*n* = 9), breast cancer (*n* = 4), pancreatic cancer (*n* = 3), carcinoma of unknown primary (CUP; *n* = 2), duodenal cancer (*n* = 1), gallbladder cancer (*n* = 1), endometrium cancer (*n* = 1), non-small cell lung cancer (*n* = 1) and renal cell cancer (*n* = 1). As expected, the survival of ovarian cancer patients was significantly longer than that of patients with non-ovarian cancer (*p* < 0.001) with a median survival time of 61 days and 362 days, respectively. The mean patients' age at time of first catumaxomab infusion was 59.6 years.

Nine patients showed significant levels of sEpCAM in ascitic fluid before catumaxomab treatment. The mean value was 4.5 ng/mL (range 1.0–11.7 ng/mL). The mean number of catumaxomab administrations in sEpCAM-positive and sEpCAM-negative patients was 3.6 infusions for both groups (Table 1). By Chi Square test we compared sEpCAM levels to age and sex (Table 1). No correlation between these clinical features and EpICD expression was found.

**Table 1:** Clinicopathological data of the study cohort

	sEpCAM ELISA in Ascites					
	Total Patients (*n*)	Negative (*n*)	%	Positive (*n*)	%	
	66	57	**86.4**	9	**13.6**	*p-value*
**Sex**						
Male	6	4	**66.7**	2	**33.7**	0.14*
Female	60	53	**88.3**	7	**11.7**	
**Age at diagnosis**						
≤60 years	34	31	**91.2**	3	**8.8**	0.24*
>60 years	32	26	**81.3**	6	**18.7**	
**Type of Cancer**						
Epithelial Ovarian Cancer	43	37	**86**	6	**14**	0.92*
Non-Ovarian Cancer	23	20	**87**	3	**13**	
**Mean number** of Catumaxomab infusions	66	3.6		3.6		0.99**
**Mean days** to next puncture	66	215		359		0.47**

* χ^2^ test

** Students *t* Test

### Correlation of sEpCAM with survival data

**Figure 1:**
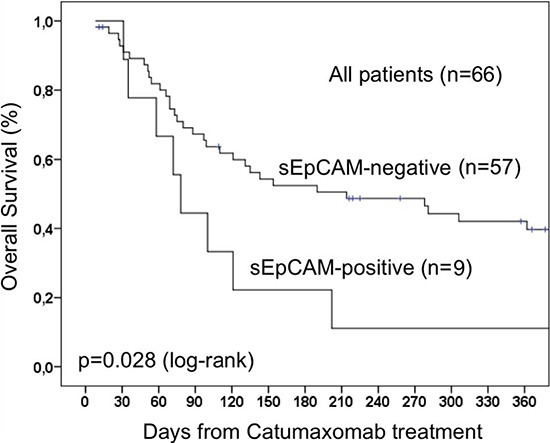
Overall survival of 66 patients treated with catumaxomab The presence of sEpCAM leads to a shorter overall survival due to neutralization effect of sEpCAM on catumaxomab.

**Figure 2:**
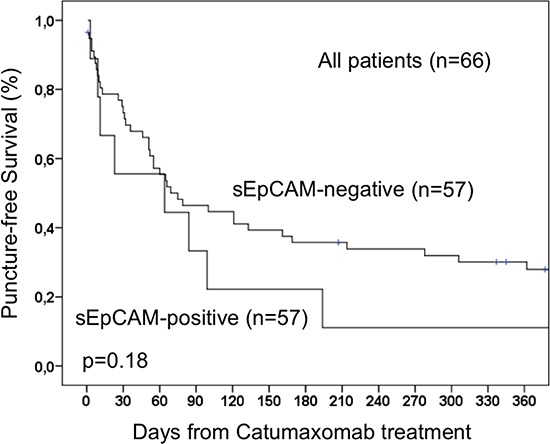
Puncture-free survival of 66 patients with malignant ascites treated with catumaxomab

**Figure 3:**
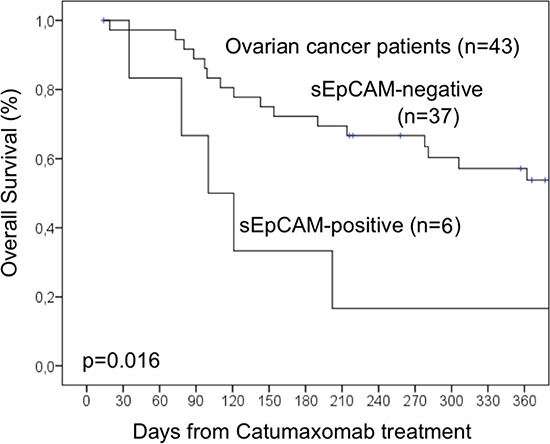
Overall survival analysis of a subgroup of 43 ovarian cancer patients

To evaluate a potential correlation of sEpCAM in ascites with the survival of catumaxomab-treated patients, Kaplan-Meier analysis and the Log-Rank test for censored survival data was applied. Of note, patients with sEpCAM-positive ascites showed a significantly poorer overall survival (*p* = 0.028; Figure 1) but no significant differences in puncture-free interval (*p* = 0.18; Figure 2). Subgroup analysis revealed even higher significance in the subgroup of ovarian cancer patients (*n* = 43, *p* = 0.016, Figure 3).

Median overall survival time for patients with sEpCAM-positive ascites was 76 days compared to 214 days in patients with sEpCAM-negative ascites.

Regarding time-to-next puncture, the median survival for patients with sEpCAM-negative ascites was not reached. For this reason, mean time to next puncture values were calculated and were 215 days and 359 days for patients with sEpCAM-positive and sEpCAM-negative ascites, respectively (*p* = 0.47).

## DISCUSSION

This is the first report describing a target-based prognostic biomarker for patients treated with the EpCAM-specific antibody catumaxomab. Up to now, there are different potential predictive markers that might be useful to select patients for catumaxomab treatment. Ott et al. described that patients that develop humoral response to catumaxomab mostly benefit from catumaxomab treatment [13]. However, this information is available only after treatment and as such may not be useful for treatment selection. In line with these observations, immunology-based biomarkers are being increasingly investigated and especially T-cell activation markers are being proposed to be tested for this purpose [14]. Data on prognostic clinical markers in catumaxomab patients were presented at ASCO meeting 2013, showing performance status, distant metastases and total serum protein being predictors of overall survival (ASCO 2013 Abstract #3078).

This study was designed to explore the predictive value of sEpCAM in ascites of catumaxomab-treated patients. A significant difference between patients with sEpCAM-positive and sEpCAM-negative ascites was observed for overall survival but not for time-to-next puncture and puncture-free survival. These observations might be explainable by a mere prognostic impact of EpCAM expression in human malignancies, which we have already observed in different previous studies. In fact, the prognostic value of EpCAM expression is still a matter of debate. We and others could observe that EpCAM overexpression is associated with poor clinical outcome in the vast majority of epithelial cancers [15–18], while few additional reports correlated EpCAM expression to good prognosis [19–21]. In breast cancer, the prognostic impact of EpCAM expression depends on breast cancer subtype [22]. In line with these somewhat conflicting clinical data, the *in vitro* effects of EpCAM on the behaviour of cancer cells seem to depend on the cancer phenotype [23] and subtypes, suggesting that the molecular role of EpCAM is context-dependent. But taken together, there remains convincing evidence that targeting the EpCAM structure might have therapeutic benefits and not disadvantages in most patients with epithelial cancers. Hence, the observation that sEpCAM-positive patients do apparently not benefit so much from EpCAM-directed catumaxomab treatment is somewhat unexpected. Most patients that participated in catumaxomab studies were tested for membrane-bound EpCAM-positive cancer cells in ascitic fluid [10]. The cancer cells of a part of these patients might shed soluble EpEX or full-length EpCAM in the ascitic fluid reflecting probably a particularly aggressive disease course that is no more revertible by catumaxomab. On the other hand, soluble EpCAM might also compete with the effector mechanisms mediated by catumaxomab. As observed by our recent *in vitro* data, recombinant EpCAM-enriched cell culture medium, with similar concentrations than those reached in ascites, counteracts the ADCC effects of catumaxomab on HEK transfected EpCAM cancer cells showing that high amounts of sEpCAM in ascites impede the full effect of catumaxomab by saturating its EpCAM-binding Fab fragment [12]. So far, to best of our knowledge, no approved antibody was described to be blocked by a soluble variant of the targeted protein.

It is tempting to suggest, that sEpCAM-positive patients could benefit from higher catumaxomab doses or more frequent applications to clear sEpCAM from the ascites to fully develop the anti-cancer mode of action of the drug.

The rate of sEpCAM-positive patients of 13.6% in this study was rather low. As observed in a previous analysis on archived ascites samples of untreated patients sEpCAM rates in patients with positive cytology are much higher (39%) [12]. Differences might be attributable to different stages of diseases. For this reason, much greater patient cohorts with fresh ascites samples have to be tested prospectively to corroborate this finding and to better define the patient population that might be excludable with this screening test.

In serum of cancer patients, sEpCAM has been described by different groups. Petsch and coworkers showed variable amounts of EpEX in the serum of cancer patients and supposed that these quantities may not interact with systemically administered therapeutic EpCAM antibodies [24]. Much higher concentrations have been described by Schmetzer *et al*. ranging to a maximum of 36 μg/mL in cancer patients [25]. These discrepant data might be attributable to different storage times of probes and consequent protein degradation, different quantification of recombinant protein controls and different binding sites of the diagnostic antibodies (full-length EpCAM *vs*. EpEX). Nevertheless, it is conceivable that these different amounts of serum sEpCAM might pass through the peritoneal barrier and accumulate in the peritoneal cavity to even higher concentrations in patients with malignant ascites driven by the high oncotic pressure. Similar concentrations as for sEpCAM were described for the soluble VEGF protein in ascites [26] but, in contrast to the observations made with catumaxomab, binding of VEGF by bevacizumab seems to positively impact the course of the disesase [27].

Taken together, soluble EpCAM in ascites appears to be a predictor of poor survival in cancer patients with malignant ascites possibly due to the neutralization effect on catumaxomab. We propose to perform prospective studies with large patient cohorts and control groups measuring sEpCAM in ascites samples to screen for catumaxomab treatment to confirm our findings.

## MATERIALs AND METHODS

### Patients and specimens

This retrospective analysis was approved by the local ethic comities of Merano (I) and Berlin (G). Written informed consent from the patients to perform analysis on ascites samples were obtained at the time of treatment start. Archived malignant ascites specimens from 66 cancer patients treated with catumaxomab during the period between December 2007 and September 2012 were acquired retrospectively. All the ascites samples were sampled before treatment start. Samples were centrifugated at 2,000 × g for 10 minutes to separate cellular components from the fluid. Supernatants were then stored at −35°C.

### EpCAM-specific Enzyme-Linked Immunosorbent Assay (ELISA)

For the detection of EpCAM in ascites we used a human EpCAM sandwich ELISA system which we had validated on its specificity and validity elsewhere [12]. Briefly, a 96-well plate was coated with a mouse anti-EpCAM monoclonal antibody overnight at room temperature in PBS pH 7.2 – 7.4. After wash steps with 0.05% Tween^®^ 20 in PBS the plate was blocked with Reagent Diluent (1% BSA in PBS, pH 7.2 – 7.4, catalog number: DY995) for 1 hour at room temperature. As positive control recombinant human EpCAM was used. Standard and supernatants were diluted in reagent diluent and incubated for 2 hours at room temperature. After washing steps, a goat polyclonal anti-EpCAM antibody was diludet in Reagent Diluent and added to each well and incubated for another 2 hours at room temperature. After repeated washing steps, streptavidin-HRP was added to the 96-well plate and incubated in the dark for 20 minutes at room temperature. After a further washing step, Substrate Solution (1:1 mixture of H_2_O_2_ and tetramethylbenzidine, catalog number: DY999) was added to each well and incubated in the dark for another 20 minutes at room temperature. A stop solution consisting in 2 N H_2_SO_4_ was used. Colour development was detected at a wavelength of 450 and 570nm using microplate reader (TECAN, Infinite F50). A seven point standard curve was used to calculate the amount of EpCAM (pg/ml) in ascites samples.

### Statistics

Statistical analysis was performed by L.B. For this purpose Statistical Package for the Social Sciences (SPSS, Chicago, IL, USA) version 11.5 was used. Chi-square and log-rank test for censored survival data was used to evaluate our results. *P*-values under 0.05 were defined as statistically significant. Time to next puncture was defined as the time span between the last day of catumaxomab infusion (day 11) and the day of next puncture. If the patient did not perform any paracentesis after treatment, the day of last visit or death was used and patients were censored at this time point. For puncture-free survival, the same time spans were evaluated as for time to next puncture but death and paracentesis after catumaxomab treatment were calculated as events. The team that performed EpCAM ELISA was unaware of clinical outcome of the patients.
